# Predictive value of matrix metalloproteinase-9 combined with magnetic resonance spectroscopy for delayed cerebral edema after spontaneous intracerebral hemorrhage

**DOI:** 10.3389/fphys.2025.1681290

**Published:** 2026-02-18

**Authors:** Jing Sun, Lei Chen

**Affiliations:** 1 Department of Imaging, Shanghai Panoramic Cloud Medical Imaging Diagnosis Center, Shanghai, China; 2 Department of Imaging, Shanghai YangZhi Rehabilitation Hospital (Shanghai Sunshine Rehabilitation Center), School of Medicine, Tongji University, Shanghai, China

**Keywords:** spontaneous intracerebral hemorrhage, delayed cerebral edema, matrix metalloproteinase-9, magnetic resonance spectroscopy, predictive value, N-acetylaspartate/creatine values

## Abstract

**Objective:**

The aim of this paper was to evaluate the predictive value of matrix metalloproteinase (MMP)-9 combined with magnetic resonance spectroscopy (MRS) for delayed cerebral edema after spontaneous intracerebral hemorrhage (SICH).

**Methods:**

Patients with head computed tomography (CT) suggestive of SICH were retrospectively included. Serum MMP-9 levels were measured within 24 h of the onset of the disease, and MRS was performed on day 3 after admission; N-acetylaspartate/creatine (NAA/Cr) values of edematous areas in MRS were measured and calculated separately. Delayed cerebral edema was defined as a 1-cm increase in the diameter of the peripheral edema around an intracerebral hematoma within 14 days compared with the peripheral diameter of the edema at 7 days, as confirmed by dynamic CT. Demographics of the delayed cerebral edema group were compared with those of the control group, along with baseline clinical data. Multivariate logistic regression analysis was applied to evaluate independent predictors of delayed cerebral edema. The predictive value of MMP-9 and MRS-related indices for delayed cerebral edema was assessed using receiver operating characteristic (ROC) curves.

**Results:**

Eighty SICH patients were included: 27 in the delayed cerebral edema group and 53 in the non-delayed cerebral edema group (control group). Univariate analysis revealed higher MMP-9 levels and NAA/Cr values in the delayed cerebral edema group relative to the control group (both *P* < 0.05). Multivariate logistic regression analysis disclosed that increased MMP-9 levels (OR = 1.041, 95% CI: 1.019–1.064, *P* < 0.001) and decreased NAA/Cr values (OR = 0.095, 95% CI: 0.015–0.586, *P* = 0.011) were independent predictive factors for the development of delayed cerebral edema after SICH. ROC curve analysis reflected that the area under the curve (AUC) of serum MMP-9 and NAA/Cr values alone and the area under the curve of the combination of the two indices for predicting the development of delayed cerebral edema after SICH were 0.835, 0.734, and 0.874, respectively.

**Conclusion:**

The combination of serum MMP-9 detection and MRS has a high efficacy in predicting the occurrence of delayed cerebral edema in SICH, providing guidance for subsequent clinical diagnosis and treatment.

## Introduction

Intracerebral hemorrhage (ICH) accounts for a quarter of all strokes worldwide and approximately half of stroke-associated mortality and disability (Planton et al., 2017). In addition to patient characteristics such as age and preexisting disabilities (including cognitive impairment) that cannot change prognosis, clinical presentation and hematoma size are also crucial prognostic factors for ICH (Veltkamp and Purrucker, 2017). Spontaneous ICH harbors a high incidence and mortality, which brings huge economic burden to medical care and social services (Akhigbe et al., 2015). ICH leads to secondary cerebral edema and injury, which may result in death and disability (Zhang et al., 2006). Cerebral edema is a frequent complication of diverse neurological diseases and a powerful predictor of prognosis, particularly in large hemispheric infarction and traumatic brain injury (Pergakis et al., 2021). The consequences of cerebral edema can be fatal, including cerebral ischemia due to impaired local or global cerebral blood flow and intracranial ventricular shift due to differential intracranial pressure, which can lead to compression of vital brain structures (Raslan and Bhardwaj, 2007). Although the burden of this disease is widely recognized, effective interventions to improve clinical outcomes are scarce. However, emerging medical and surgical choices also exhibit promising effects on functional outcomes (Al-Kawaz et al., 2020).

Cranial computed tomography (CT) is the preferred imaging method for diagnosing acute ICH. Some CT parameters can forecast hematoma enlargement and neurological deterioration. These CT parameters and clinical standards have been utilized to create practical scoring schemes for predicting hematoma enlargement (Garg and Biller, 2019). Magnetic resonance spectroscopy (MRS) is a useful non-invasive tool used to characterize biomarkers of biological processes, which extends traditional magnetic resonance imaging through offering an additional dimension of spectral information depicting the abnormal existence or concentration of metabolites of interest (Laruelo et al., 2013). CT and MRI methods have the disadvantages of being bulky and unable to supply bedside and emergency on-site monitoring. In addition, these devices are very expensive, which limits their utilization in economically underdeveloped areas (Pan et al., 2015). Matrix metalloproteinases (MMPs) are a group of zinc-dependent endopeptidases that are able to degrade components of the extracellular or neurovascular matrix, contributing to neurovascular damage (Rosenberg, 2009). MMP has been demonstrated to participate in the formation and development of brain edema, along with multiple molecular interactions (Yang et al., 2016). MMP-9 belongs to the MMP family, which has a degrading impact on body’s vascular basement membrane, promotes neutrophil migration, exacerbates local inflammation, ultimately destroys the cerebral vascular barrier, and further induces cerebral edema (Yang et al., 2016). MMP-9 functions in the acute phase after ICH, during which restriction of MMP activity or elimination of the *MMP-9* gene has been revealed to have the potential to treat subsequent brain injury (Wang and Tsirka, 2005). Mouchtouris et al. (2021) has stated that patients with hypertensive ICH who experience neurological symptoms within 24 h have elevated MMP-9 levels, which are positively associated with the edema degree around the hematoma and patients with consciousness disorders. Based on this, this study was conducted to evaluate the predictive value of MMP-9 plus MRS for delayed cerebral edema post-SICH.

## Materials and methods

### Ethical approval

The study was approved by the Ethics Committee of Shanghai Panoramic Cloud Medical Imaging Diagnosis Center. Written informed consent was obtained from all participants.

### General information

Eighty patients with SICH indicated by head CT admitted to our hospital from July 2021 to March 2024 were selected. The study included patients ① aged 18–80 years; ② who had their first onset of illness within 24 h of symptom onset and were clearly diagnosed through head CT, with a hospitalization period of ≥2 weeks; ③ who had not received any relevant medication or surgical treatments prior to admission to the hospital; ④ who underwent at least three head CT examinations in the period of 24 h to 9 days and 12–21 days after the onset of illness; and ⑤ who signed an informed consent form. The exclusion criteria were ① patients with severe liver and kidney insufficiency, serious infection, tumor, and other malignant diseases; ② patients with secondary cerebral hemorrhage; ③ patients who required surgical treatment, died during hospitalization, or discontinued treatment; ④ patients whose cerebral hemorrhage broke into the ventricle and could not calculate the amount of hemorrhage; ⑤ patients who had combined subarachnoid and epidural hemorrhage; and ⑥ patients with incomplete clinical data.

### Treatment

In accordance with the relevant guidelines, all patients received dehydrating agents to lower intracranial pressure; effective control of blood pressure, blood glucose, and nutritional support for nerve health; prevention of pulmonary infection and stress ulcers; and other related conservative internal medicine treatment and routine care (Al-Kawaz et al., 2020).

### Diagnostic criteria of delayed cerebral edema

The patients were admitted to the hospital and gradually relieved after 7 days of treatment, but their condition deteriorated from the 7th to 14th day. The hematoma was absorbed to a certain extent as observed in the repeated head CT, but there was an extensive edema band around the hematoma focus, and the volume of the edema and the occupying effect were increased compared with those before the treatment. The patients were classified into the delayed cerebral edema group (27 cases) and the non-delayed cerebral edema group (the control group, 53 cases) accordingly (Cai et al., 2019).

### Clinical data collection

Clinically relevant data of patients were harvested, including gender, age, past medical history, baseline blood pressure, baseline hematoma volume, bleeding site, and Glasgow Coma Score (GCS) (Healy et al., 2019). Baseline hematoma volume and bleeding site were assessed based on CT findings on the day of patient admission.

### MMP-9 measurement

Enzyme-linked immunosorbent assay (ELISA) was utilized for examining MMP-9 levels, and the MMP-9 ELISA kit was procured from ADL, United States. In brief, 6 mL of fasting venous blood was obtained from all patients on the next day of admission, and the prepared specimens were placed in the standard wells and sample wells of the enzyme plate, followed by 2-h incubation at 37 °C. The liquid in each well was removed, and 100 μL detection solution A was sequentially added to each well. After 1 h of incubation at 37 °C, the solution was removed, followed by the addition of 100 μL detection solution B. After another 1 h of incubation at 37 °C, 90 μL enzyme labeling reagent was added to each well. After replacing the microplate sealers, the solution was incubated at 37 °C for 15–25 min, followed by the addition of 50 μL of the termination solution, and the optical density value at wavelength 450 nm was read to obtain the final value of MMP-9 protein concentration (Bollmann et al., 2021).

### MRS detection

A two-dimensional multivoxel chemical shift spectroscopy imaging method was applied, with point-resolved MRS sequences, and the region of interest (ROI) was selected to be acquired with point-resolved spin-echo spectroscopy in the region of perihematoma edema on T2-weighted imaging (T2WI). Compounds analyzed through spectroscopy included N-acetylaspartate (NAA) and creatine (Cr), and NAA/Cr values were recorded (Rhodes, 2017).

### Statistical analysis

Data were processed using SPSS 27.0 software. Normally distributed measurement data were reported as mean ± standard deviation (mean ± SD) and processed using the t-test; non-normally distributed measurement data were indicated as median and interquartile spacing [M (IQR)] and processed using the rank-sum test. Categorical data were represented as the number of cases and percentage (n, %), and the χ^2^ test was utilized. Independent predictors were analyzed using a multivariate logistic regression model. Receiver operating characteristic (ROC) curves were calculated as the area under the curve (AUC). *P* < 0.05 was considered a statistically significant difference.

## Results

### General information

In total, 134 patients with acute cerebral hemorrhage within 24 h of onset were admitted during the study period, and 80 patients with SICH were finally enrolled in the analysis following the inclusion and exclusion criteria. The age of the patients ranged from 29 to 89 years; 52 cases were male, and 28 cases were female. There were 27 cases in the delayed cerebral edema group and 53 cases in the non-delayed cerebral edema group (control group). Univariate analysis revealed that there were no differences between the two groups in terms of age, gender, past medical history, baseline blood pressure, baseline hematoma volume, bleeding site, and GCS (all *P* > 0.05; Table 1).

**TABLE 1:** Comparison of general information between the two groups [mean ± SD, n, M (IQR)].

Item	Control group (n = 53)	Delayed cerebral edema group (n = 27)	χ^*2*^ */t/z*	*P*
Age (years)	60.23 ± 10.09	63.81 ± 8.60	−1.579	0.119
Gender				
Male	35 (66.04)	17 (62.96)	0.074	0.785
Female	18 (33.96)	10 (37.04)		
Past medical history				
Diabetes	15 (28.30)	8 (29.63)	0.015	0.901
Hypertension	33 (62.26)	17 (62.96)	0.004	0.951
Baseline blood pressure				
Systolic pressure (mmHg)	170.45 ± 25.95	177.52 ± 31.16	−1.075	0.286
Diastolic pressure (mmHg)	92.87 ± 11.67	95.30 ± 12.34	−0.863	0.391
Baseline hematoma volume (mL)				
10∼19	16 (30.19)	8 (29.63)	0.075	0.963
20∼29	26 (49.06)	14 (51.85)		
30∼40	11 (20.75)	5 (18.52)		
Bleeding site				
Cerebral lobe	15 (28.30)	7 (25.93)	0.051	0.822
Deep brain	38 (71.70)	20 (74.07)		
GCS	13.00 (2.00)	14.00 (1.00)	−1.739	0.082

### Values of MMP-9 levels and MRS-related indices

Then, the values of MMP-9 levels and MRS-related indices were compared between the two groups, which indicated higher MMP-9 levels in the delayed cerebral edema group than in the control group (179.99 ± 39.18 vs. 138.00 ± 28.62), and meanwhile, the NAA/Cr values in the delayed cerebral edema group were notably lower than those in the control group (1.59 ± 0.27 vs. 1.90 ± 0.41) (both *P* < 0.05, Table 2).

**TABLE 2:** Comparison of the values of MMP-9 levels and MRS-related indices between the two groups (mean ± SD, n).

Group	MMP-9 (mg/L)	NAA/Cr
Control group (n = 53)	138.00 ± 28.62	1.90 ± 0.41
Delayed cerebral edema group (n = 27)	179.99 ± 39.18	1.59 ± 0.27
*t*	−5.430	3.497
*P*	<0.001	<0.001

### Analysis of risk factors for delayed cerebral edema after SICH

Multivariate logistic regression analyses were implemented using the variables with *P* < 0.05 in Tables 1 and 2 (MMP-9 and NAA/Cr) as independent variables and the occurrence of delayed cerebral edema during hospitalization as the dependent variable. Elevated MMP-9 levels (OR = 1.041, 95% CI: 1.019–1.064, *P* < 0.001) and decreased NAA/Cr values (OR = 0.095, 95% CI: 0.015–0.586, *P* = 0.011) were independent predictors of the development of delayed cerebral edema after SICH (Table 3).

**TABLE 3:** Analysis of risk factors for delayed cerebral edema after spontaneous cerebral hemorrhage.

Variable	*β*	*S.E*	*Wald*	*P*	OR	95% CI	
						Lower limit	Upper limit
MMP-9	0.041	0.011	13.874	<0.001	1.041	1.019	1.064
NAA/Cr	−2.355	0.929	6.426	0.011	0.095	0.015	0.586

### Analysis of the predictive value of MMP-9 levels and MRS-related indices alone or in combination for delayed cerebral edema

**FIGURE 1:**
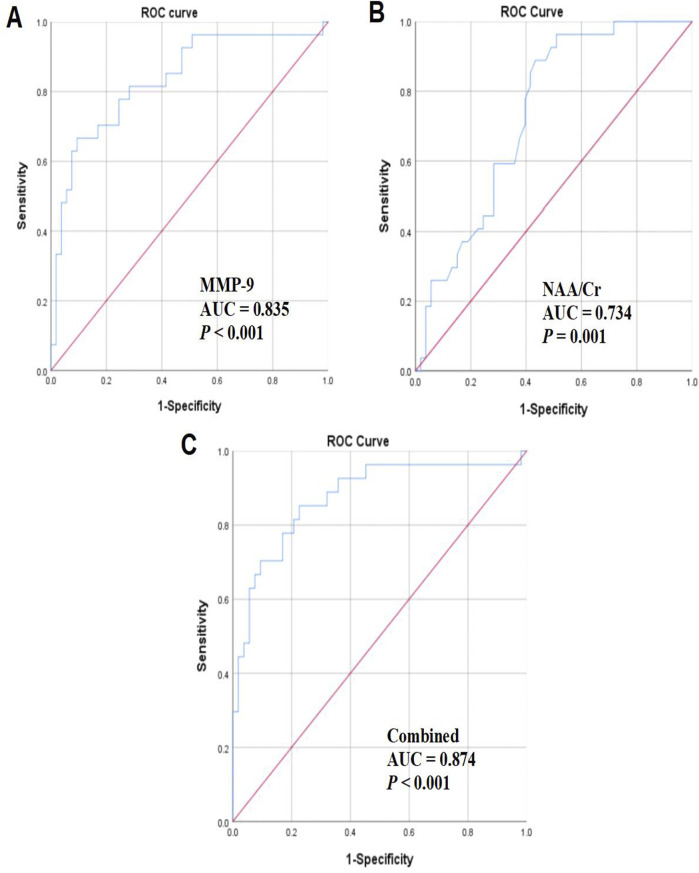
Analysis of the predictive value of MMP-9 levels and MRS-related indices alone or in combination for delayed cerebral edema. **(A)** Predictive value of MMP-9 levels; **(B)** predictive value of NAA/Cr; **(C)** predictive value of MMP-9 levels in combination with NAA/Cr.

We then assessed the predictive value of MMP-9 levels and MRS-related indicators for delayed cerebral edema. The findings unveiled that the AUC of MMP-9 was 0.835 (*P* < 0.001, Figure 1A), with an optimal cutoff value of 0.573, a sensitivity of 66.70%, and a specificity of 90.16%. The AUC of NAA/Cr was 0.734 (*P* = 0.001, Figure 1B), with an optimal cutoff value of 0.455, a sensitivity of 88.90%, and a specificity of 56.60%. The AUC for the joint analysis was 0.874 (*P* < 0.001, Figure 1C), with an optimal cutoff value of 0.626, a sensitivity of 85.20%, and a specificity of 77.40% (Table 4; Figure 1).

**TABLE 4:** Analysis of the predictive value of MMP-9 levels and MRS-related indices alone or in combination for delayed cerebral edema.

Variable	AUC	*P*	Optimal cutoff value	Sensitivity (%)	Specificity (%)	95% CI	
						Lower limit	Upper limit
MMP-9	0.835	<0.001	0.573	66.70	90.16	0.736	0.934
NAA/Cr	0.734	0.001	0.455	88.90	56.60	0.627	0.842
Combined	0.874	<0.001	0.626	85.20	77.40	0.786	0.963

## Discussion

SICH from multiple sources can result in transient mass effect, cause damage to the surrounding brain, and often lead to early neuronal death. Hematoma may contribute to secondary brain injury, neurological deficits, and occasionally delayed death if the patient survives the initial event (Xi et al., 2004). Although the latest advances in SICH treatment have notably reduced mortality rates, no intervention measures so far have markedly improved functional recovery (Kim et al., 2016). Hematoma compresses and destroys brain tissue, leading to primary brain injury. Inflammation and coagulation reactions post-ICH can accelerate the formation of perihematoma brain edema, leading to more severe and persistent damage (Zheng et al., 2016). In view of this, this study was conducted to evaluate the predictive value of MMP-9 plus MRS for delayed cerebral edema post-SICH.

MRS is a non-invasive and non-ionizing technique that can measure metabolic turnover rates and *in vivo* tissue concentrations of more than 20 metabolites and compounds in the human central nervous system (Henning, 2018). In clinical applications, MRS is able to potentially distinguish primary brain tumors from other potential mimetics, including demyelinating diseases, infections, or lymphomas (Weinberg et al., 2021). Current limitations of MRS include limited resolution for metabolites that resonate in a narrow spectral range (e.g., glutamine and glutamate) and the inability to determine low concentrations of metabolites (Grover et al., 2006). MMP is a zinc-dependent protease generated by astrocytes and endothelial cells, and the primary role of MMP-9 is to degrade extracellular matrix components (You et al., 2019). MMP-9 is involved in blood–brain barrier disruption in the process of hemorrhagic transformation and aggregates brain damage after cerebral ischemia (Tang et al., 2004). MMP-9 levels are lower in blood and brain tissue under normal conditions, and its levels are significantly elevated in the presence of tumors, inflammation, and other pathological conditions within the body. MMP-9 expression has been indicated to be upregulated in brain tissues of ICH patients, and hydrolyzed laminin and collagen fibers lead to the disruption of cellular tight junctions and extracellular matrix molecules, along with the increase in cerebral vascular barrier permeability, which promotes the occurrence of cerebral edema (Esfandiari et al., 2019; Pasi et al., 2019). There are several possible mechanisms of MMP-9 in secondary injury post-ICH: disruption of the extracellular matrix and basement membrane, activation of vascular endothelial growth factor and thrombin, and apoptosis (Chang et al., 2014). Nevertheless, there are no studies examining the potency of MMP-9 combined with MRS in diseases, particularly cerebrovascular-related diseases.

Decreased NAA/Cr reflects neuronal damage and necrosis in edematous regions, which is consistent with histologic observations of varying degrees of necrosis and apoptosis in edematous regions, and NAA/Cr is a vital index for MRS analysis. The results of our paper revealed that the NAA/Cr value of the edema region was reduced in patients with delayed cerebral edema post-SICH. The NAA/Cr value can reflect the occurrence of delayed cerebral edema. By analyzing the changes in the NAA/Cr ratio in MRS, the occurrence of delayed cerebral edema can be detected in time, thus providing evidence-based basis for further treatment. Kobayashi et al. (2001) showed a sustained decrease in NAA/Cr in the surrounding area of ICH at 48 h and 2 weeks, with a negative correlation between NAA/Cr and hematoma volume. In addition, we observed that increased MMP-9 levels and decreased NAA/Cr values were independent predictive factors for the development of delayed cerebral edema after SICH, and the ROC curve analysis reflected that MMP-9 levels and MRS-related indices alone or in combination showed a high diagnostic value for delayed cerebral edema. However, the combination of the two has the strongest diagnostic effect.

In summary, this study underscores that the combination of serum MMP-9 detection and MRS has a high efficacy in predicting the occurrence of delayed cerebral edema in SICH, providing guidance for subsequent clinical diagnosis and treatment. Further large-scale, multicenter studies are warranted to confirm these findings.

## Data Availability

The original contributions presented in the study are included in the article/supplementary material, further inquiries can be directed to the corresponding author.
